# Squamous cell carcinoma of the posterior pharyngeal wall: A comparative analysis of oropharyngeal origin versus hypopharyngeal origin

**DOI:** 10.1017/S002221512400224X

**Published:** 2025-07

**Authors:** Petr Daniel Kovarik, Jakub Cvek, Rahul Patil, Charles Kelly, Malcolm Jackson, Laura MacKenzie, Nick West, Nicholas Willis, Josef Paul Kovarik, Muhammad Shahid Iqbal

**Affiliations:** 1 Department of Trauma and Orthopaedics, Northumbria Specialist Emergency Care Hospital, UK; 2 Northern Centre for Cancer Care, Newcastle upon Tyne, UK; 3 Department of Oncology, University of Ostrava, Ostrava, Czech Republic; 4 Institute of Dentistry and Oral Sciences, Palacky University Olomouc, Olomouc, Czech Republic

**Keywords:** posterior pharyngeal wall, head and neck cancer, radiotherapy, dysphagia

## Abstract

**Background:**

The posterior pharyngeal wall is an anatomical subsite of both the oropharynx and hypopharynx. The treatment outcomes of squamous cell carcinoma (SCC) of these sites are generally published together, which makes the interpretation of data challenging. The aim of this analysis was to determine if there is any difference in the treatment outcomes of these two rare disease entities.

**Materials and Methods:**

Retrospetive analysis showed that the posterior pharyngeal wall was the primary subsite in 17 patients (1.65 per cent) out of 1031 patients with oropharyngeal SCC, and in 23 patients (11.73 per cent) out of 196 patients with hypopharyngeal SCC.

**Results:**

The five-year overall survival was 45 per cent for oropharyngeal origin and 53 per cent for hypopharyngeal origin patients. There was no significant difference in survival and locoregional control between these two groups of patients.

**Conclusion:**

Squamous cell carcinoma of the posterior pharyngeal wall is a rare entity, which in our series represents 1.65 per cent of oropharyngeal cases and 11.73 per cent of hypopharyngeal tumours. There was no difference in treatment outcomes between the two groups.

## Introduction

The posterior pharyngeal wall is an anatomical subsite of both the oropharynx and hypopharynx. At the oropharyngeal level, it extends from the level of the junction of the hard and the soft palate free border superiorly to the level of the hyoid bone inferiorly. At the hypopharyngeal level, it extends from the level of the hyoid bone (level of the body of the third cervical vertebrae) down to the oesophagus (level of intervertebral discs 6 and/or 7 vertebrae).

Primary squamous cell carcinoma (SCC) of the posterior pharyngeal wall of the oropharynx is a rare entity, scientific literature is sparse and it is not clear what proportion of oropharyngeal SCC this subsite represents. The majority of SCC involving the posterior oropharyngeal wall originate in the nearby sites, usually tonsil and soft palate, and directly extend to this structure.^1^ For outcomes of treatment of primary SCC of the posterior pharyngeal wall of the oropharynx as a sole subsite or posterior pharyngeal wall, specifically arising in the hypopharynx, there is a paucity of information in the existing literature.

Similarly, SCC of posterior pharyngeal wall in the hypopharynx is also rare.^2^ Treatment outcomes for SCC of the posterior hypopharyngeal wall remains challenging, with 5-year survival of 50 per cent, even in patients with early-stage disease (T1 and/or T2).^2^^,^^3^

Because of the rarity of this disease, the outcomes of treatment of these sites (posterior pharyngeal wall of the oropharynx and hypopharynx) are generally published together, which makes the interpretation of the data more challenging.^4^^–^^7^

The primary aim of this analysis was to determine the treatment outcome of these rare disease entities with the aim of determining if there are any difference in the outcomes of the two subgroups (oropharyngeal origin versus hypopharyngeal origin).

## Materials and methods

From January 2010 to December 2022, 1031 patients with oropharyngeal SCC and 196 patients with hypopharyngeal SCC were treated with radical or adjuvant radiotherapy (RT) and/or chemoradiotherapy at a single oncology centre. Out of 1031 patients with oropharyngeal SCC, the posterior pharyngeal wall was the primary subsite in 17 patients (1.65 per cent) and out of 196 patients with hypopharyngeal SCC, the posterior pharyngeal wall was the primary subsite in 23 patients (11.73 per cent). These 40 patients with primary SCC of the posterior pharyngeal wall were the subjects of this further analysis.

All patients had staging investigations which included computed tomography of the neck and thorax and/or magnetic resonance imaging of the neck to delineate the extent of the tumour. In all patients histological confirmation of SCC was obtained. As per institutional policy, all patients were discussed at the local multidisciplinary team meetings comprising head and neck surgeons, clinical oncologists, pathologist, radiologist, cancer nurse specialists, dietician and speech therapist, and the management plan was agreed.

Patients were treated with radical dose of RT consisting of 65–66 Gy given in 30 daily fractions or 55 Gy given in 20 daily fractions. This short regimen of 20 fractions was employed for logistic reasons during the coronavirus disease 2019 pandemic. A dose of 60 Gy in 30 daily fractions was used in the adjuvant setting. Concerning the treatment volume, the gross tumour volume encompassed the whole primary tumour and involved lymph nodes with a 1-cm margin to obtain the clinical target volume (CTV65/66). The remainder of the involved lymphatic level was treated with 60 Gy (CTV60). The uninvolved neck was treated with a prophylactic dose of 54 Gy in 30 daily sessions. Dose distribution was calculated using the planning systems OncoCentra Masterplan, Accuray TomoTherapy or Raystation. Until 2013, the patients were treated with three-dimensional (3D) conformal RT, following which patients were treated with intensity-modulated RT.

After treatment completion, patients were followed up at three-monthly intervals for the first two years, then every six months for three years by the head and neck surgical team. Survival intervals were defined as the time between the beginning of the treatment (surgical resection or first day of RT) and occurrence of the first event. Late effects of the treatment were classified according to Common Terminology Criteria for Adverse Events v5.0.^8^

Statistical analysis was performed in GraphPad Prism 9 (GraphPad, Boston, USA). Kaplan–Meier survival curves were created and the log-rank test was used to determine statistical significance (*p* < 0.05).

This retrospective study was registered with the local hospital effectiveness register as a part of a service review project (No: 14533). The data used in this study were obtained from the previous clinical practice and the patients’ identification and personal information was protected anonymously. The authors followed the institutional ethical protocols.

## Results

The characteristics of the patient cohort are summarised in Table 1.

**Table 1.** Characteristics of the patient group

Characteristic	Oropharynx (*n* (%))	Hypopharynx (*n* (%))
Total number (all subsites)	1031	196
Posterior pharyngeal wall	17 (1.65%)	23 (11.73%)
Age (median (range); years)	65 (54−77)	69 (58−74)
Gender		
Male	11	17
Female	6	6
Smoking		
Non-smoker	2	3
Current smoker	8	9
Past smoker	5	10
Unknown	2	1
T stage		
1	1	1
2	5	9
3	8	10
4	3	3
N stage		
0	9	10
1	2	3
2	5	9
3	0	1
p16 and/or HPV status		
Positive	1	0
Negative	13	0
Unknown	3	23
Intent of the treatment		
Radical	16	21
Adjuvant	1	2
Treatment modality		
3D CRT	4	5
IMRT	13	18
Concomitant chemotherapy (cisplatin)		
Yes	8	13
No	9	10
Dose fractionation of RT		
65−66 Gy in 30 daily fractions	15	18
60 Gy in 30 daily fractions	1	2
55 Gy in 20 daily fractions	1	3

HPV = human papillomavirus; 3D = three-dimensional; CRT = conformal radiotherapy; IMRT = intensity-modulated radiotherapy; RT = radiotherapy

### Squamous cell carcinoma of the posterior wall of the oropharynx

Out of 17 patients with primary posterior oropharyngeal wall SCC, four patients were treated using 3D conformal RT (until 2013) and following that all patients were treated using intensity-modulated RT (*n* = 13). One patient underwent radical surgery followed by adjuvant RT and the remaining 16 patients were treated with radical RT and/or chemoradiotherapy. Sixteen patients had a 6-week RT regimen (65–66 Gy in 30 daily fractions in a radical setting and 60 Gy in 30 fractions in the adjuvant setting) and 1 patient received a radical regimen of 55 Gy in 20 daily fractions (Table 1).

The median follow up of all patients was 24 months (range, 3–85 months). At the time of analysis, eight patients had died: three from cancer and five from other causes (cancer free). Two patients developed local recurrence alone; one died of progressive disease. Neither of the two patients who experienced local recurrence alone were deemed suitable for salvage surgery. Two patients developed local recurrence in combination with distant metastases (DM). One of these two patients developed local recurrence and lung metastases, and has died, whilst the other patient developed local recurrence and bone metastases, and is alive and receiving immunotherapy. Two patients developed distant metastases only without local recurrence; one developed lung metastases and died from disease, and one developed bone metastases and is alive. In total, local recurrence without distant metastases developed in 4 patients (23.5 per cent) at a median of 4 months (range, 2–8 months) and distant metastases developed in 4 patients with or without local recurrence at a median time of 7.5 months (range, 2–17 months).

The p16 status was negative in 13 patients, positive in 1 patient and not available in 3 patients. The only patient with p16 positivity was a 54-year-old male with T1 N0 M0 disease treated with radical resection followed by adjuvant RT. This patient remains well, with no signs of recurrence, 58 months after the treatment.

Radiotherapy treatment was associated with significant late toxicity with dysphagia. In 13 evaluable patients, 3 were asymptomatic (grade 0), there was grade I dysphagia in 6 patients, grade II in 1 patient, 2 patients remained gastrostomy tube dependent (grade III) and in 1 patient complete stenosis developed, requiring salvage laryngo-pharyngectomy (grade IV) (Table 2).

**Table 2.** The incidence of late toxicity (dysphagia) according to the National Cancer Institute Common Toxicity Criteria Scale version 2.0 for pharyngeal dysphagia related to radiation^8^

	Oropharynx (*n* = 17)			Hypopharynx (*n* = 23)		
Late toxicity (dysphagia)	Total	3D CRT	IMRT	Total	3D CRT	IMRT
Evaluable patients (*n*)	13	3	10	12	3	9
Grade 0 (none)	3	1	2	3	0	3
Grade I (mild dysphagia, but can eat regular diet)	6	0	6	1	0	1
Grade II (dysphagia, requiring predominantly pureed, soft or liquid diet)	1	1	0	2	0	2
Grade III (dysphagia, requiring feeding tube, intravenous hydration or hyperalimentation)	2	1	1	5	3	2
Grade IV (complete obstruction – cannot swallow saliva; ulceration with bleeding not induced by minor trauma or abrasion or perforation)	1	0	1	1	0	1
Grade V (death related to toxicity)	0	0	0	0	0	0

3D = three-dimensional; CRT = conformal radiotherapy; IMRT = intensity-modulated radiotherapy

### Squamous cell carcinoma of the posterior hypopharyngeal wall

Out of 23 patients with posterior hypopharyngeal wall SCC, five were treated using 3D conformal RT and 18 had intensity-modulated RT. Radical RT and/or chemoradiotherapy was given to 21 patients and the remaining 2 patients were treated in an adjuvant setting. Three patients were treated with a regimen of 55 Gy in 20 daily fractions and the remaining 20 patients had 6 weeks of RT. The median follow up of all 23 patients was 41 months (range, 5–137 months). Fifteen patients died: nine from their cancer and six from non-cancer causes. The median follow up of the eight surviving patients was 57 months (range, 6–137 months).

Two patients were treated in an adjuvant setting. The primary tumour had been resected completely in both patients followed by 60 Gy given in 30 fractions. One patient died of recurrent disease 42 months after RT and the second patient died cancer-free 106 months after RT. Distant metastasis developed in neither patient.

Local recurrence developed in 8 patients (34.7 per cent), with a median interval of 20 months (range, 7–88 months). Distant metastases developed in 3 patients, with a median time of 16 months (range, 11–20 months), and in 2 of these there was also local recurrent disease.

Similarly to oropharyngeal posterior pharyngeal wall disease, treatment of the hypopharyneal posterior pharyngeal wall was associated with significant late dysphagia. Late toxicity with dysphagia was evaluable in 12 out of 23 patients (in the excluded 11 patients, local recurrence developed in 8 patients and 3 patients were diagnosed with a second primary in the oesophagus during their follow-up period). Out of 12 evaluable patients, 1 was asymptomatic (grade 0), there was grade I dysphagia in 1 patient, grade II in 2 patients and 5 patients remained gastrostomy tube dependent (dysphagia grade III). In one patient stenosis developed requiring surgical intervention; this patient also remained gastrostomy tube dependent (dysphagia grade IV). There was no grade V toxicity (Table 2).

**Figure 1.**
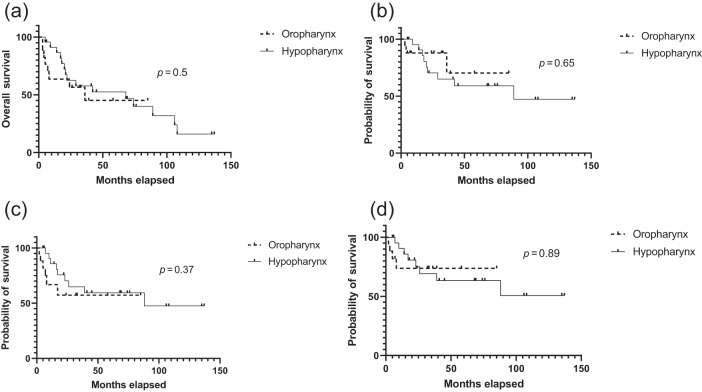
Treatment outcomes: (a) Overall survival, (b) disease-specific survival, (c) progression-free survival and (d) locoregional control.

The outcomes of treatment are summarised in Figure 1 and a late dysphagia toxicity comparison is made in Table 2. The median overall survival times for posterior pharyngeal wall tumours of oropharynx and hypopharynx origin were 36 and 68 months, respectively. The 5-year overall survival was 45 per cent for oropharyngeal origin patients and 53 per cent for hypopharyngeal origin patients. There was no statistically significant difference in overall survival (*p* = 0.5), disease-specific survival (DSS; *p* = 0.65), progression-free survival (*p* = 0.37) or locoregional control (*p* = 0.89) between the two groups of patients (Figure 1).

## Discussion

Most of the published studies consider the posterior pharyngeal wall to be a single structure and do not distinguish between oropharyngeal and hypopharyngeal origin. This is mainly because SCCs located at the posterior pharyngeal wall often overlap and it can be difficult to identify the exact origin. Anatomically, the oropharynx and hypopharynx are different sites and the National Comprehensive Cancer Network strictly distinguishes the posterior pharyngeal wall of the oropharynx from that of the hypopharynx.^9^

Squamous cell carcinoma of the posterior pharyngeal wall is a rare entity. A large systematic review of the outcome of different treatment strategies for the SCC of the posterior pharyngeal wall was published in 2020 and included 11 studies (534 patients in total).^10^ This review provided useful information on treatment outcomes; primary surgery followed by adjuvant RT appeared to provide the best outcomes, especially for locally advanced cases, but the studies reviewed did not provide information on whether they were the posterior pharyngeal wall of oropharyngeal or hypopharyngeal origin. Also, there was some bias in the studies.

The difference in T-stage and overall stage of the disease between patients treated with primary surgery (*n* = 150, 6 studies), primary RT (*n* = 145, 3 studies), and combined surgery and RT (*n* = 239, 2 studies) was not specified. Another source of bias could be the difference in the quality of the treatment. Most patients treated with RT (66 per cent) were treated before 2000, in comparison with 17 per cent of patients who were treated with primary surgery in the same period. Also, there was no explicit information on the details of RT delivery (e.g. dose and/or fractionation, RT modality, i.e. 3D conformal RT and intensity-modulated RT) and the potential impact of these variables on the treatment outcomes. The lack of information concerning the treatment technique and dose and/or fractionation is particularly important as one study suggested that hyperfractionated RT treatment might have an impact on local control.^11^

Published in 2016, De Felice *et al*.^12^ compared the outcome of surgical and radiation treatment of patients with posterior pharyngeal wall tumours. Nine patients were treated with surgery alone, 46 with surgery followed by adjuvant RT, 23 patients with surgery followed by adjuvant chemoradiotherapy and 11 with neoadjuvant chemotherapy followed by surgery and adjuvant chemoradiotherapy (total *n* = 89 treated with surgery and/or adjuvant RT and/or chemoradiotherapy). In the non-surgical group (*n* = 91), 13 patients received neoadjuvant chemotherapy followed by definitive chemoradiotherapy, 28 had definitive RT alone and the remaining 50 patients had definitive chemoradiotherapy. The authors concluded that surgery followed by RT provided better outcomes than non-surgical treatment. For the whole cohort, 5-year overall survival was 33.4 per cent (43 per cent for surgical and/or adjuvant treatment and 24.1 per cent for the non-surgical group, *p* = 0.02). However, it is important to note that there were more early-stage (T1 and/or T2) cases in the surgical group and there were more advanced T-stage cases in the non-surgical group, and this difference was significant (*p* = 0.01).

Multivariate analysis showed that smoking, T3 and/or T4 disease, well-differentiated SCC and non-surgical treatment were associated with reduced survival. However, subgroup analysis showed that the survival benefit of surgical treatment was limited only to patients with T1 or T2 but not T3 and T4 disease. The authors stated that definite RT and/or chemoradiotherapy was generally considered when the patients were medically unsuitable for surgery or when the tumours had no clear limits. This study provided useful information on the two subsites, i.e. oropharyngeal and hypopharyngeal origin posterior pharyngeal wall. There were 51 per cent patients with oropharyngeal origin and 49 per cent patients with hypopharyngeal origin. The primary origin (oropharyngeal versus hypopharyngeal) was not a prognostic factor for overall survival (*p* = 0.7), progression-free survival (*p* = 0.8) and locoregional control (*p* = 1.0).

In our study, 37 out of 40 patients had definitive RT and/or chemoradiotherapy and only 3 patients had surgery followed by adjuvant RT. Sixty per cent of patients (24 out of 40) had an advanced T-stage (T3 and/or T4) disease. There was no difference in overall survival between oropharyngeal and hypopharyngeal origin patients and our study supports the presumption that although these are two different entities, there is no difference in the outcome of the treatment. It may be that the locoregional recurrence occurs earlier in oropharyngeal posterior pharyngeal wall than in the hypopharyngeal posterior pharyngeal wall (median 4 months *vs* 20 months), but this is observation rather than statistically significant evidence.

The late toxicity of RT of the posterior pharyngeal wall is well recognised, and our data are supportive of this. Dysphagia is a dominating symptom. Descriptively, RT of the posterior pharyngeal wall of the hypopharynx appears to be associated with a higher frequency of severe dysphagia than RT at oropharyngeal posterior pharyngeal wall level. In our series, 6 out of 23 patients (26 per cent) with a hypopharyngeal posterior pharyngeal wall developed severe late dysphagia (grade III or higher) as compared with 3 out of 17 patients (17.6 per cent) with an oropharyngeal posterior pharyngeal wall.

As far as the impact of p16 and/or human papillomavirus (HPV) status in the primary SCC of the posterior pharyngeal wall is concerned, although tumour–node–metastasis-8 includes all p16 positive oropharyngeal primaries, there is evidence that the prognostic value of p16 in other oropharyngeal subsites (excluding tonsils and base of the tongue) is questionable. In our study, there was only one patient with p16 positive status therefore its impact is not evaluable. Marklund *et al*.^13^ analysed the impact of p16 status and concluded that p16 status should be evaluated only in oropharyngeal SCC of the tonsils or base of the tongue primary tumours. Other oropharyngeal subsites should be staged as HPV-unrelated (p16 negative) SCC.

There are certain limitations of this study. Firstly, this is a retrospective study and thus may carry selection bias, although every effort was made to minimise this. All consecutive patients treated with radical and/or adjuvant intent were included in the analysis. Secondly, given the low number of patients, a meaningful multivariable comparative analysis was not possible, but given the rarity of disease, it is important to publish studies even with low numbers of patients, which can add information to the existing literature.

- The incidence of posterior pharyngeal wall primary was 1.65 per cent in oropharynx and 11.73 per cent in hypopharynx patients
- The 5-year overall survival was 45 per cent for oropharyngeal origin disease
- The 5-year overall survival was 53 per cent for hypopharyngeal origin disease
- There was no difference in survival and locoregional control between the two origins of disease

## Conclusion

Squamous cell carcinoma of the posterior pharyngeal wall is a rare entity; in our series it represented 1.65 per cent of all oropharyngeal cases and 11.73 per cent of all hypopharyngeal tumours. There was no difference in treatment outcomes between the tumours of oropharyngeal origin compared with those of hypopharyngeal origin, although a trend of delayed recurrence and better median survival was observed in te hypopharyngeal posterior pharyngeal wall. These results should be treated with caution given the retrospective nature of the study and low number of patients. To guide optimal management, a multicentre collaborative approach is needed to conduct randomised controlled trials in this comparatively small patient population.
